# Scaffolding Junior Middle School Students’ Engagement in Online Project-based Learning During the COVID-19 Pandemic: A Case Study from East China

**DOI:** 10.1177/21582440221131815

**Published:** 2022-10-20

**Authors:** Cheng Zhong, Keyi Lyu

**Affiliations:** 1 Chinese University of Hong Kong, Hongkong, China; 2 Hangzhou Normal University, Hangzhou, China

**Keywords:** COVID-19 pandemic, online learning, junior middle school, student engagement, project-based learning, scaffolds, mixed methods

## Abstract

During the COVID-19 pandemic, online learning has experienced increasing utilization and poses new challenges for schoolteachers to engage students. Project-based Learning (PBL) is widely acknowledged as an effective pedagogy for motivating and involving students. However, few studies have examined scaffolds that facilitate student engagement in the context of distance PBL. This mixed-method study was conducted with grade 7 teachers and students in a junior middle school in East China from March 2020 to April 2020. Qualitative analysis was employed in interviews with 2 teachers and 21 students. Quantitative analysis was used to visualize the self-reflection reports of 39 students. The findings suggest that the scaffolds of teacher direction, technology support, peer collaboration, and parental assistance play a significant role. In addition, specific scaffolding within the above categories was revealed. The results highlight the problem-oriented, methodological, and synthesized application of various scaffold(ing)s in engaging students and emphasize that scaffolding students emotionally is the core issue to support engagement for remote learning.

## Introduction

During the COVID-19 pandemic, global governments were forced to shut down the assembly of a large number of people, including the K-12 schools (kindergarten through 12th grade schools). Most countries have turned to online learning mandates to avoid the disruption of learning as much as possible (UNESCO, 2020).

While distance education serves as a practical and promising alternative for teachers and students, it also challenges teachers in maintaining and promoting students’ active engagement in technology-mediated learning circumstances (Huang, Tlili et al., 2020). A large-scale online survey conducted with 2,401 primary and secondary school teachers in China indicated that 62.6% and 52.1% of teachers felt that it was challenging to organize instruction activities and to interact with students online, respectively (Wang, Wang et al., 2020). A survey from the United States also suggested that teacher respondents need support to involve students in distance learning (Hamilton et al., 2020).

Previous studies have illustrated a variety of pedagogies, tenets, and tools for reaching a high level of student engagement (Bender, 2005; Dixson, 2010; Henrie et al., 2015; Tay et al., 2021; Zepke & Leach, 2010). Project-based learning (PBL), a student-centered pedagogy, is one of the most effective forms of instruction (Budner & Simpson, 2018; Kay et al., 2000). But it is remarkable that students are not necessarily active participants in PBL. Rather, Bate et al. (2014) articulate that students’ active involvement and sense of responsibility for learning is paramount to implementing PBL (see also, Chiu & Hew, 2018). Engaging students in PBL is no less than challenging (Tambouris et al., 2012). For instance, in a SWOT analysis of PBL, Cho and Brown (2013) argue that students could be easily distracted in the PBL process. Lopez-Gazpio (2022) argues that PBL is a challenge for both teachers and students as PBL implies changes of roles, instruction and learning practices, and evaluations.

Hence, the questions of “how to engage students *through* PBL” and “how to engage students *in* PBL” should be addressed simultaneously. While the first question identifies PBL as a useful pedagogy for engaging students’ online learning, the second requires investigating the scaffolds for PBL to engage students. Previous studies have generated rich discussions of the principles and strategies of conducting online PBL. For instance, Koh et al. (2010) propose four guidelines for implementing online PBL, including assigning students a suitably complex problem, structuring project milestones, inspiring students to articulate their learning through project materials, and assessing students’ knowledge construction. Ravn Haslam et al. (2021) highlight that belonging to a group can promote students’ online engagement and suggest conducting group-based activities and introducing social collaboration tools.

However, few studies have examined the scaffolds and scaffoldings for online PBL. Generally, scaffolding is a form of timely external support that pedagogically propels students to a higher level (Gonulal & Loewen, 2018). This study understands scaffolding as a form of opportune support that promotes students’ engagement in online PBL. Based on a PBL project conducted in a middle junior school in East China from March to April 2020, this study aims to decipher the typologies of scaffolding and seeks to understand how different scaffold(ing)s work together to improve or impede student engagement in online PLB learning.

In addition, this study reverses prior research reasoning, which engages in using technology to facilitate pedagogies in online PBL. By drawing on new technologies and flexible technology usage, the past research contributes to innovative pedagogies. However, they also led to the misty jungle of pedagogy. Rather than suggesting new appropriate pedagogies, this study integrates technology into pedagogical inquiry (Baran et al., 2011) and examines technology as a pedagogy in its own right.

## Theoretical Background

### Engaging Students in Online PBL: Facilitators and Scaffold(ing)s

Project-based learning is based on the capability of engaging students. The above argument is not groundless, but is based on a plethora of research that emphasizes PBL’s engaging nature. For instance, Blumenfeld et al. (1991) define project-based learning as “a comprehensive approach designed to *engage* students in investigating authentic problems.” The Buck Institute for Education also uses the lexicon “engagement.” As noted, project-based learning is “a systemic teaching method that engages students in learning knowledge and skills …”(2003). Others, such as Solomon (2003), also argue that project-based learning is designed to engage students (See also, Carrabba & Farmer, 2018; Robinson, 2013).

While previous studies claim that PBL can engage students, they also admit that students do not naturally engage in PBL. Recent studies have highlighted the role of technology in supporting online PBL. For example, Kokotsaki et al. (2016) elaborate on how digital technology facilitates PBL implementation. According to them, technology can help students document and share their procedural achievements and final creations.

Focusing on the higher education phase, Bond and Bedenlier (2019) provide a bioecological framework of student engagement, which posits technology as a potential facilitator. They illustrate various technological factors that affect student engagement, such as access to technology, technology choice, and technology usability (Bond & Bedenlier, 2019). Specifically, they suggest that students will be more engaged in the contexts in which they use familiar technologies and have knowledge of the technology they use. Further, educational technologies can enhance student engagement by endowing students with a sense of autonomy and developing a positive online learning environment with a high level of belonging (Bedenlier et al., 2020).

In addition to technology, other facilitators of teachers, parents, and peers have been discussed (Skinner & Pitzer, 2012). Kwok and Tan (2004) developed a collaboration model for PBL that involves teachers, students, parents, and subject experts. In their model, teachers are the main facilitators who introduce and use pedagogical scaffolds to support collaborative learning in PBL. Subject experts have become a scaffold for inspiring students’ thinking. Emphasizing peer collaboration in project-based learning, Pazos et al. (2019) argue that balanced team participation, shared goals, and clear roles are crucial scaffolding. Egenrieder (2010) argues that like teachers, parents can also facilitate students’ interest and investment in PBL. Parents can participate in PBL and create new peer relationships as informal educators. In addition, Egenrieder warns that parents’ excessive participation may decrease students’ self-efficacy.

As the above analysis demonstrates, teachers, parents, peers, experts, and technology can facilitate the provision and application of scaffolds for online PBL. Moreover, these facilitators themselves can serve as scaffolding that engages students. Because this study involves teachers, parents, peers, and technology, it develops these facilitators into four categories of scaffolds.

### Indicators of Student Engagement: Cognition, Behavior, and Emotion

Regarding the indicators of student engagement, most studies follow Astin’s (1984) classification of cognitive, behavioral, and emotional engagement (Burch et al., 2015; Malan, 2020; Redmond et al., 2018). For example, Chapman (2003) established three-dimensional criteria (cognitive, behavioral, and affective) to measure students’ engagement levels in specific tasks. Behavioral engagement refers to students’ involvement in project activities based on their participation and persistence. Cognitive engagement indicates the students’ willingness and ability to undertake learning tasks. Affective engagement refers to students’ feelings during the project process (Chiu, 2021). Notably, in some recent studies, agentic engagement was added to the indicator list (Chiu, 2022; Reeve, 2013). Agentic engagement refers to students’ proactive actions, suggesting that students recognize what they want and the need for learning (Reeve, 2013). There is no doubt that agentic engagement is a crucial indicator; however, unlike the other dimensions, students’ agency plays both a causal and effectual role in engagement. Hence, this study did not consider agentic engagement as an indicator.

### Developing a Tentative Model of Scaffolding Student Engagement in Online PBL

**Figure 1.**
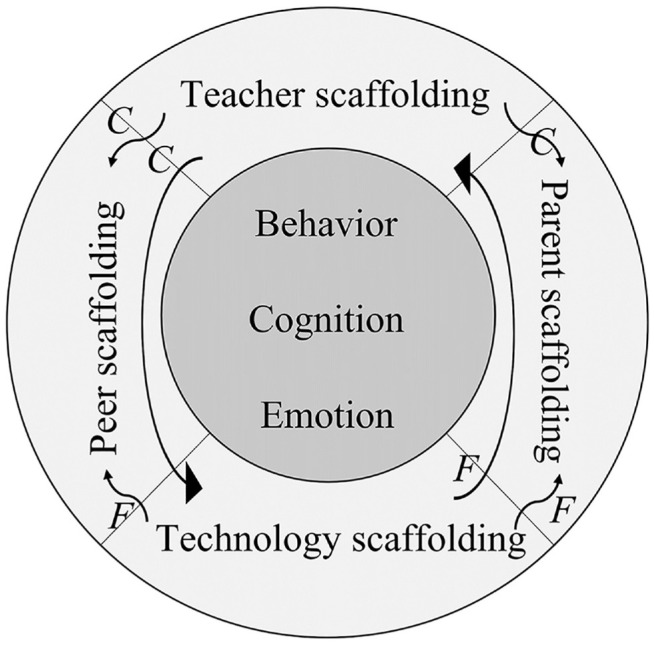
The tentative model of scaffolding student engagement in online PBL. *Note*. C means controls. F means facilitate.

Based on the above analysis, a tentative model for scaffolding student engagement in online PBL was developed (See Figure 1).

As Figure 1 illustrates, this study temporally identified four scaffolds for engaging students in online PBL. This model posits that teachers play an initial leadership role by controlling the operation of other scaffolds to ensure that they promote student engagement (Mergendoller et al., 2006). The technology scaffold enhances the efficiency of the other scaffolds.

## Methodological Notes

### The Context of this Study

In China, nationwide distance learning during school closure is referred to as a large “experiment” that tests the application and efficiency of technology-mediated teaching and learning (Liang et al., 2020). As a part of the big experiment, this study was initially supported by the Specific Project of Zhejiang Province Office for Education Science Planning (from 2020 to 2021).

First, it aimed to explore the pedagogy of online PBL. During the research process, the authors further focused on scaffolds that promote students’ engagement. The research question is as follows:

1. What are the specific typologies of teachers, parents, peers, and technology scaffold(ing)s that engage students in online PBL?
2. How do these scaffold(ing)s work together to promote or impede students’ engagement in online PBL?

The authors comprise the principal investigator and participants of the project. The research project was conducted in Grade 7 in a middle school in East China. The school is an elite private school, where most students are from middle to upper class families and have computers and networks at home. From March 2020 to April 2020, the authors conducted two consecutive online PBL projects at the school. Four Grade 7 teachers were invited to conduct these projects. One hundred seventeen students participated in the learning project. The first learning project directed students to develop and negotiate urgent policies to slow the spread of pandemics at an early stage. The second project encouraged students to participate in a mini-debate on social topics relevant to the COVID-19 pandemic. The first project was selected as the case study. Teachers, students, and parents were informed of their rights and signed consent forms before participating. The study was conducted according to the guidelines of the Declaration of Helsinki, and approved by the Zhejiang Province Office for Education Science Planning on Human Research Project (protocol code 2020YQJY419).

### Project Design

The project featured in the case study for this paper is titled *Fighting Against the Unknown Virus*. The project assignment was to develop appropriate policies to control the spread of the pandemic while ensuring the continuation of different living demands. Students in each class were randomly divided into six groups and played the roles of policymakers and policy reviewers. Policymakers worked in groups to collect information, discuss, and design public policies against unknown viruses. The remaining students took the role of policy reviewers, who were required to imagine themselves as people from all walks of life (i.e., teachers, doctors, nurses, lawyers, school-aged children, parents, café owners, blue-collar workers, etc.) and to challenge policymakers’ proposals. Policymakers submitted the final version of their policies after debates with the policy reviewers.

The project included three stages. Stage 1 was the launch stage, during which: (1) The teacher introduced the project assignments and rubrics. (2) The teacher introduced the online platform to be used for conducting PBL—the Learning Circle in DingTalk—which consisted of sections, including notice, Q & A, and sharing. The teacher placed the project assignments, rules, and rubrics in the notice section for students’ reference. In the sharing section, the teacher uploaded the materials and links needed by the students to accomplish their projects. (3) The teacher created five new sections for each governmental group, issuing their policies and negotiating with the reviewers.

Stage 2 was the product-creation stage, during which the teacher checked the project progress and managed the time. The teacher also responded to students’ inquiries regarding technology use., completed the performative assessment, and provided timely feedback to the students. Students used Groupchat Box and Learning Circle to conduct discussions and debates, respectively. Groupchat Box facilitated students’ creation of a chat group for instant communication and resource sharing.

Stage 3 was the assessment stage, during which the teacher documented each student’s performance and delivered individual assessments. Students were required to conduct mutual assessments of their peers in addition to a self-assessment.

### Data Collection and Analysis

This study adopts an exploratory sequential mixed method to collect and integrate qualitative and quantitative data (Berman, 2017; Guest, 2012; Plano Clark et al., 2008). A QUAL-quant method design feeds qualitative findings for quantitative visualization and interprets the quantitative figures using qualitative analysis (Guest, 2012). The process was divided into two stages. First, two teachers (T1 is a 33 years-old male; T2 is a 29 years-old female) and 21 students attended the interview. Second, 39 students submitted a self-report. The demographic information of the student participants is presented in Table 1.

**Table 1.** Demographic Information of Student Participants.

Demographic information of student participants								
	Age (years old)			Gender		Online learning experience (years)		
	13	14	15	male	female	1	2–3	≥4
Interviewee	13	7	1	11	10	3	14	4
Self-report provider	22	15	2	21	18	7	24	8

#### Stage 1: Interview and qualitative thematic analysis

The teacher interviews explored how teachers facilitate students’ behavioral, affective, and cognitive engagement through online PBL design and implementation. The teachers shared the tools and strategies they use to engage students in different online learning steps. They were also asked to discuss challenges and countermeasures. Students were asked to talk about what and how various scaffold(ing)s facilitate or impede their online engagement. To ensure that the interview was grounded in research questions and a theoretical framework, the researchers tactically encouraged students to talk about the relationship between their engagement (cognitive, emotional, and behavioral) and the scaffolds of teachers, peers, parents, and technology.

The teacher interviews lasted 40 to 50 minutes, and the student interviews lasted 25 to 40 minutes. All the interviews were conducted in Chinese and transcribed into English. The translations were double-checked by the authors.

Qualitative thematic analysis was then adopted to identify various scaffoldings (Braun & Clarke, 2006; Judger, 2016). The following sequential steps were followed: (1) the authors read and familiarized themselves with the interview; (2) the authors generated initial codes independently; (3) the authors compared and merged their initial codes; (4) the authors derived themes from the initial codes and then cross-checked their analysis; (5) the authors reviewed, negotiated, identified, and defined the themes; and (6) the authors developed a five-point Likert scale (see Table 2) based on the themes and categories that emerged from the qualitative data. According to Fereday and Muir-Cochrane (2006), an iterative and reflexive process is necessary for promoting and demonstrating rigor of thematic analysis (see also, Roberts, Dowell et al., 2019). From Step 3 to 5, the authors conducted continuous comparisons, discussions and reflection on codes. In this process, the authors also invited a supervisor to code the data. The results were compared, and no further modifications were required.

**Table 2.** Sample From the 5-point Likert Scale for Students.

Section 1. Teacher scaffoldings
1. Teacher’s driving questions
1.1 How helpful are the driving questions in helping you *behaviorally* engage in online PBL?
□ Unhelpful □ Not very helpful □ Average □ Helpful □ Very helpful
1.2 How helpful are the driving questions in helping you *cognitively* engaging in online PBL?
□ Unhelpful □ Not very helpful □ Average □ Helpful □ Very helpful

#### Stage 2: Self-report and quantitative visualization

Self-reporting is widely employed to evaluate student engagement (Chapman, 2003). Thirty-nine students submitted a self-report form using a five-point Likert scale (see Table 2). Students were asked to access the extent to which scaffolding facilitates their online engagement.

To ensure the validation of the survey, the meanings of “behaviorally engage,”“cognitively engage,” and “emotionally engage” were explained to students to ensure their understanding. The behavioral aspect involves peer discussion, interaction with policymakers/policy reviewers, concentration on tasks, progress, and time management. The cognitive aspect involves self-efficacy, online resource search strategies, policy design, policy defense, policy critics, policy revision, and reflection. The emotional aspect involves items such as liability, cheering, enjoyment, safety, and a sense of group.

The quantitative data were processed using the following steps. First, “unhelpful” counted as 1 point, “not very helpful” as 2 points, “average” as 3 points, “helpful” as 4 points, and “very helpful” as 5 points. Second, the average number of points for each scaffolding was determined as it denotes the strength of the different scaffoldings. For example, in Stage 1, the strength of scaffolding of driving questions counted as point 4.5, 4.7, and 4.7 points in facilitating behavioral, cognitive, and emotional engagement, respectively. Finally, Excel was used to visualize the strength and dynamics of the scaffold(ing)s.

## Findings

Through thematic analysis, which underpins this study’s tentative of scaffold(ing) model frame (see Figure 1), the tentative categories of scaffolds were specified as follows: teacher direction, peer collaboration, parent assistance, and technological support. In addition, corresponding scaffoldings emerged from the data. This section illustrates the strengths and applications of various scaffoldings in the aforementioned categories.

### The Teacher-Direction Scaffold and Its Scaffoldings

When describing how teachers scaffold online student engagement, teachers and students used a variety of words, including “direction,”“navigation,”“guide,”“lead,” and “supervision.” By carefully reading and contrasting the interview texts, this study settled on using the term *teacher direction* to shed light on teachers’ continuous and robust control over project objectives, assignments, and progress (Bouchard, 2009).

As one of the students stated:

*The teacher is an online game designer, and we are the players. He set up goals, rules, and rubrics. Though you cannot see him, you know he is there.*

**Figure 2.**
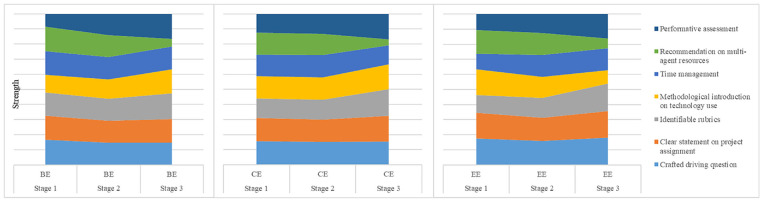
Dynamics of teacher-direction scaffoldings. *Note*. BE indicates behavioral engagement. CE refers to cognitive engagement. EE refers to emotional engagement. Strength refers to the capacity for scaffolding to support students’ online engagement.

There are seven scaffoldings in the teacher-direction category (see Figure 2): (1) crafted driving question, (2) clear statement on project assignment, (3) identifiable rubrics, (4) methodological introduction to technology use, (5) time management, (6) highly-recommended resources, and (7) performance assessment.

#### Crafted driving question

The crafted driving question facilitates continuous and powerful online student engagement (see Figure 2). In this case, it is described as:

*In the context of the early spread of the unknown virus, how can policies slow pandemics and protect various stakeholders’ interests?*

According to the students, the crafted driving question scaffolds them by arousing a sense of responsibility. Keeping the concept of saving others’ lives in mind, students actively participate and constantly devote themselves to the project. According to the students, while the driving question brings them a sense of duty, it also causes them to feel nervous and stressed. Students state that the project is “so real” as it models what is genuinely happening. Students stated,

*In this project, I am no more a student but an official with power. Every decision I make is related to the life of ordinary people (Said by a student who performed the policymaker).*

*I shall be very careful. I must find every statement in the policy that has the potential to cause harm to the life of ordinary people (Said by a student who performed the policy reviewer).*

#### Project assignment statement, identifiable rubrics, and time management

While the crafted driving question evokes students’ emotions and further impacts their behavior, the teacher’s statement on project assignments and rubrics directly guides students to implement the project tasks. One student stated,

*The assignments tell me what to do and the rubrics tell me how to deal with the assignments. Also, the teacher helped us to check the progress.*

As aforementioned, while the crafted driving question endowed students with a sense of responsibility, it also makes students nervous. Teachers’ clear statements on project assignments and rubrics reassured students and improved students’ self-efficacy in accomplishing projects.

#### Methodological introduction on technology use

Although most students had online learning experiences, two-thirds were using DingTalk for the first time. Hence, students appreciated the teacher’s introduction to technology use and argued that the teacher’s instruction in technology facilitated their effective use of DingTalk. One student stated,

*I have heard of DingTalk. I thought it was just a messaging app like WeChat and MSN. Through teacher’s introduction, I learned that this app has multiple functions to assist learning.*

Notably, teachers’ introduction of technology was not merely about how to use the platform. The teacher also elaborated on how technologies, such as the Learning Circle and Groupchat Box, can help students. The teacher’s introduction was as follows, “In the Learning Circle, we work together to form a community. We speak of our voices, share resources, and exchange ideas. Challenges and criticism from others are valuable.”

The teacher’s account presented the online learning platform as more than a tool or learning method. Adopting the metaphor of community, teachers united students with common interests, thereby slowing the spread of the virus. Learning Circle technologies have become a methodology for conducting student work during project implementation. Students argued that teachers’ descriptions of technology strengthened their sense of community and facilitated their group work. One student articulated,

*Teacher’s introduction promotes my understanding of “learning circle.” I realize that we are more than a group. Precisely, we are a community and the Learning Circle is the base of our community. We sit in a circle and share a common goal. It is everyone’s responsibility to have their own voice to improve the products.*

#### Performative assessment

At the launch stage, students generally felt stressed when confronted with performative assessments. However, as the project progressed, students established the knowledge regarding the necessity of performative assessment because indicators such as attendance rate, times of speaking, and discussion were not involved in the assessment. Instead, the teachers observed the group chat and praised students’ insightful ideas, shared high-quality resources, and practical communication skills. Facilitation of performative assessment thus changed from creating pressure to enhancing cognition. Students also argued that the performative assessment allowed them to reflect on their performance and adjust their ways of participating in the project in a timely manner.

#### Recommendation of multi-agent resources

The teacher’s recommendation of resources strongly supported student engagement in Stages 1 and 2. Notably, teachers’ recommendation of resources was not limited to online resources, such as news, databases, and governmental websites. Teachers, parents, peers, groups, and the self were also described as resources. According to the teacher, during the project, the teacher can guide and assess students’ performance. parents can provide suggestions for designing and reviewing policies. parents can also help students effectively communicate with your group. Students should help each other. The teacher argued that the most powerful resources for students are the students themselves.

Recognizing the multiple agents as resources, students felt more supported. One student accounted,

*Although I have experienced group activities, I always thought learning was a personal affair. Project-based learning is different from the learning activities I once attended. The power of one person is insufficient to fulfill the project. For the first time, I realized the importance of mutual help. I appreciate those who are willing to help me. I have never felt supported like this.*

According to the students, they regarded teachers and parents as supervisors rather than facilitators. Teachers’ explanations of multi-agent resources made students more willing to communicate with their teachers and parents. One student stated,

*When we were implementing learning activities at school, teachers were beside us to supervise us. When we were doing school assignments at home, parents were beside us to inspect us. I perceived teachers and parents as the supervisors who made us concentrate on our work. During the project-based learning, I found they were not supervisors but facilitators. They joined in discussion and offered suggestions.*

### The Peer-collaboration Scaffold and its Scaffoldings

**Figure 3.**
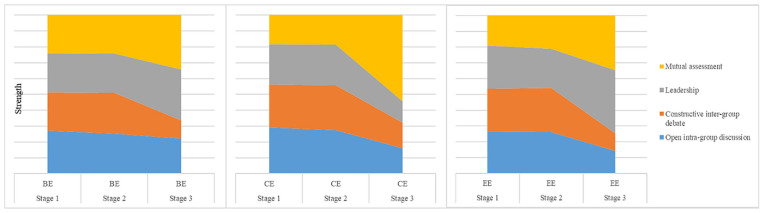
Dynamics of peer-collaboration scaffoldings. *Note*. BE indicates behavioral engagement. CE refers to cognitive engagement. EE refers to emotional engagement. Strength refers to the capacity for scaffolding to support students’ online engagement.

More than a learning outcome, peer collaboration is a pedagogical strategy (Hussein, 2021). In PBL, peer collaboration can facilitate students’ exchange of resources, ideas, experiences, and knowledge, and can promote student motivation and persistence (Koh et al., 2010; Lou & Kim MacGregor, 2004). Based on the interviews, this study derived four scaffoldings:(1) intra-group discussion, (2) inter-group debate, (3) leadership, and (4) mutual assessment of peer collaboration. (See Figure 3).

#### Intra-group discussion

Teachers provided the scaffolding of intra-group discussions in Stages 1 and 2 when the teacher encouraged the students to help each other and have discussions with one another. According to the students, intra-group discussion was an important supplement to teachers’ face-to-face pedagogical facilitation. Cognitively, students found that they could solve problems by themselves instead of relying on teachers. Students argued that intragroup discussions increased self-efficacy. One student stated,

*In the past, when I had a problem that I could not solve by myself, I turned to my teacher for help. The teacher would tell me either the answer or the ways of solving problem. In the project-based learning, I found that I can work out the solutions by discussing problems and share ideas with community members. I will no longer wait for teacher to give me an answer.*

During Stage 2, the policy reviewers’ challenges and policy designers’ criticisms made students further recognize the individual’s limitations and invoked a strong sense of belonging. One student accounted,

*I suffered at Stage 2. The challenges from policy reviewers and other policymakers are difficult to respond to. Fortunately, my group members have been with me all the time. We reflect and perfect product together.*

#### Inter-group debate

The inter-group debate in Stage 2 was essential for students to create the final policy product. Students were required to face the challenges of others from various perspectives and to propose appropriate policies. As intra-group discussion created a sense of belonging, the inter-group debate reinforced this sense.

*You can never imagine…we are like in a war. We then discuss, design, and post-policies. Then we are challenged, we go back to re-search materials, re-organize policy texts, respond to challenges…then we are rechallenged… we must mutually depend on each other.*

#### Leadership

Rather than being offered by teachers, the scaffolding of leadership was developed by students. In both face-to-face learning circumstances and a technology-mediated environment, PBL posed a high requirement for student leadership (Prince et al., 2005). Leadership plays a critical role in facilitating the implementation of student projects (Walters & Sirotiak, 2011). Because teachers, in this case, divided students into groups randomly and did not give any instruction on leadership, distinct leadership landscapes emerged among the groups. During the interview, three groups of students argued that the leadership of their groups was satisfactory. Students in one group stated the leadership was passable and the other two groups complained the leadership of their groups. According to teachers and students, groups with high-level leadership worked more efficiently and created higher-quality production than those without good leadership. High-quality leadership made group discussions and debates progress in a well-organized and orderly manner.

#### Mutual assessment

When the above three scaffoldings were withdrawn in Stage 3, the scaffolding of mutual assessment was offered. While mutual assessment promotes students’ cognitive engagement, it also affects students’ emotional engagement. Unlike the previous scaffoldings that incited students’ positive emotions, mutual assessment produced negative emotions, which however enhanced students’ cognitive engagement level. One student narrates:

*It is not easy to accept others’ criticism, especially after you devote a lot of time to accomplishing the projects. I feel sad when I see the comments from my group members…they think I am not a good communicator…I do not deny it. I feel sad, but I know it is true…I will change.*

It is worth mentioning that students’ perceptions of mutual assessment were influenced by teacher direction. Students conveyed their opinions on mutual assessment as follows:

*Mutual assessment is also a type of collaboration. We know more about what and how the group members do in the project than our teachers…During the mutual assessment, we do not communicate and cooperate as we did in the former stage. However, we still helped each other.*

### The Parent-assistance Scaffold and Its Scaffoldings

**Figure 4.**
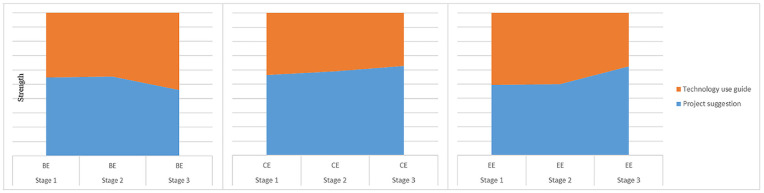
Dynamics of parent-assistance scaffoldings. *Note*. BE indicates behavioral engagement. CE refers to cognitive engagement. EE refers to emotional engagement. Strength refers to the capacity for scaffolding to support students’ online engagement.

Teachers regard parents as an essential source of learning. Parent-assistance scaffoldings strongly engaged students in Stages 1 and 2 (see Figure 4).

#### Project suggestion

As mentioned, teachers suggested that students seek help from their parents. According to the teachers’ initial plan, parents would provide students with suggestions on policy design and reviews based on their experiences. Parents were much more helpful than teachers. According to the students, parents also offered advice on intra- and inter-group communication.

In addition, parents greatly assisted students to appropriately recognize mutual assessment. One student stated,

*At the very beginning, I do not like peer assessment. I think it is unfair…My mother persuades me to treat mutual assessment more rationally. She tells me that people cannot know everything about others, and I only need to accept those that I admit. She shared her personal experiences with me.*

#### Technology use guide

The parents’ technology use guide, together with the teacher’s statement on technology use and peers’ mutual help, offered three pillars for students to overcome the technology problems. Compared to teachers and peers, parents’ guidance on technology use was more helpful for students in home-based learning. According to the students, they could participate in online learning more efficiently with their parents’ guidance on technology use. One student articulated,

*While teacher helped a lot in introducing the functions of DingTalk, my parents helped me operate the computer. For example, there seemed to be something wrong with my laptop build-in mike, my classmates could not hear my voice. I did not have a clue. My father checked the laptop and found I muted myself.*

### The Technology-support Scaffold and its Scaffoldings

**Figure 5.**
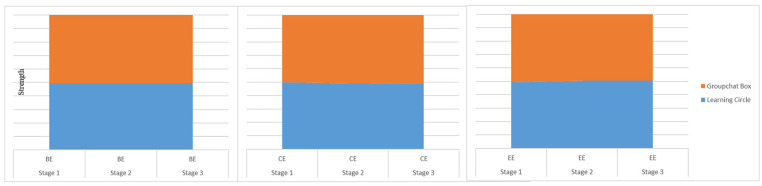
Dynamics of technology-support scaffoldings. *Note*. BE indicates behavioral engagement. CE refers to cognitive engagement. EE refers to emotional engagement. Strength refers to the capacity for scaffolding to support students’ online engagement.

It is widely acknowledged that technology can support various types of online learning, including project-based learning (Bedenlier et al., 2020; Bond & Bedenlier, 2019). Based on the teacher and student interviews, two scaffoldings were included under the category of technology support—the Learning Circle and Groupchat Box. Figure 5 presents the dynamics of these scaffoldings.

Students argued that the Learning Circle not only facilitated communication, but also changed their understanding of learning.

*In the face-to-face classroom, we followed the teacher’s instructions. For me, learning means the following. Learning circle makes me feel like a natural learner…I do not know how to describe this. It is like…you are aware that you are learning. You can understand your thinking and the learning processes.*

Moreover, during the interview, most students indicated that they “learn to learn” through Learning Circle.

*This is the first time I feel that learning must be learned. In the online project-based learning, everything is at once familiar and strange. The network-based learning environment makes me reflect my previous learning experiences. I find that there are different ways of learning. Teacher, parents, classmates and I play different roles in online PBL from those in face-to-face learning.*

According to the students, the Learning Circle was more than a learning platform. It served as a “learning model” that guided their learning behavior. Using the Learning Circle, students recognized the basic steps of self-directed learning, including identifying goals and task, collecting learning materials, and accomplishing tasks with the help of teachers and peers.

Groupchat Box is a platform for intra-group discussion among students. Similar to the Learning Circle, Groupchat Box records students’ collaboration processes and offers a platform for students to supervise each other. In addition to mutual supervision, it enables students to formulate a sense of belonging in teacher-direction, as previously mentioned.

## Discussion and Implications

As presented, the above scaffold(ing)s discussed in this study are intertwined and combine to engage students. This study also sheds light on the problem-oriented, methodological, and synthesized application of various scaffold(ing)s and emphasizes that scaffolding students emotionally is the core issue for remote learning.

Among the scaffold(ing)s, teacher-direction plays a central role, as it determines how and when to implement or remove the other scaffold(ing)s. While previous research emphasizes that teachers provide pedagogical scaffolds, the “how tos” are ignored (Ertmer & Glazewski, 2019; Ward & Lee, 2002). Resonating with the existing literature (Chung & Chow, 2004; Sihaloho et al., 2017), teacher-direction scaffold(ing)s are first oriented as problem-solving in this study. This implies that teachers must be clear about what problems should be addressed and what kind of scaffoldings should be provided in each stage. Further, because technology-mediated learning environments change in conjunction with changes in projects and specific educational technologies, teacher-direction scaffold(ing)s should offer methodologies for applying the other scaffold(ing)s. For example, in this case, teachers encouraged students to use the Learning Circle and Groupchat Box as ways of learning in a community.

The teacher-directed methodological guidance on technology use also alluded to a different understanding of technology. In previous studies, technology has been externally incorporated into PBL as a pedagogical tool (Solomon, 2003; Watson, 2002). This study’s results indicate that technologies such as the Learning Circle and Groupchat Box are more than instruments, but ways of teaching and learning. Instead of being used under certain pedagogies, they enable innovative pedagogies for PBL (Tambouris et al., 2012). For example, the Learning Circle enables the teacher to build an open discussion and co-supervise space, and offers students a pattern of cooperative learning. This does not suggest that online PBL must use the Learning Circle or Groupchat Box. Instead, the results imply that teachers should go beyond an instrumental perspective and integrate technology into pedagogy (Baran et al., 2011).

As the findings show, teachers in this study underestimated the function of the parent-assistance scaffoldings. Parents were regarded as technology user guides in the teacher’s initial plan. However, results indicate that parents facilitated students’ understanding of the significance of project tasks and promoted their emotional engagement. Thus, teachers should strengthen their cooperation with parents in online PBL. Many studies have emphasized peer-collaboration scaffold(ing)s to improve students’ autonomous learning in a face-to-face learning environment (Ching & Hsu, 2013; Hou et al., 2007). This study adds an online case to the current body of literature.

While existing studies give equal weight to students’ cognitive, emotional, and behavioral aspects, the findings suggest that emotional engagement should be given more consideration (Chiu, 2021; Jamaludin & Osman, 2014). As the findings show, scaffoldings such as driving questions, rubrics, and mutual assessment invoke students’ feelings (e.g., anxieties, worries, sense of belonging, and sense of responsibility), which strongly improve or impede students’ involvement. The findings also demonstrated that one scaffolding can simultaneously produce positive and negative feelings. For instance, when the driven question aroused students’ sense of responsibility, it also made them nervous. While previous studies advocate avoiding or erasing negative feelings (Lee et al., 2013; Lee, Huh et al., 2015), the findings suggest that the negative feelings can improve students’ engagement. For example, students’ nervousness in intergroup discussions enhanced their sense of belonging. This implies that teachers should identify students’ feelings in a timely manner and make use both negative and positive feelings to facilitate learning.

## Limitations and Suggestions for Further Research

This study has three limitations. First, it was conducted in an elite school in East China, where teachers and students are equipped with advanced technologies and relative knowledge. These findings may not be fully generalizable to developing regions. Future studies should be conducted in regions and schools that lack online teaching conditions and experiences. Second, the qual-quan research design determined that the correlation between scaffold(ing)s and students’ online engagement cannot be accurately measured. Quantitative studies should be conducted to examine the strengths and relationships between scaffoldings. Further, the correlation between the three types of engagement and scaffold(ing)s should also be examined. Third, because this study focuses on identifying the scaffold(ing)s for engaging students in online PBL, it lacks examinations of the pedagogy of employing scaffold(ing)s. A study on developing a pedagogy for using scaffold(ing)s in online PBL should be conducted. Significantly, when future studies establish pedagogies for using scaffoldings, the pedagogy should not center around the scaffold(ing)s, which will change along with the demand of students. In this sense, pedagogical innovation should focus on the how to conduct the appropriate and flexible selection and application of different scaffolds.

## Conclusion

While the principles and tenets that theoretically support PBL have been widely discussed, the scaffold(ing)s employed in practical context lacks attention (Ertmer & Glazewski, 2019; Pazos et al., 2019; van Rooij, 2009). Ertmer and Glazewski (2019) proposed two basic functions of scaffolding: supporting students in complementing complex learning tasks and engaging students in tasks. Focusing on the latter, this study examines which and how various scaffold(ing)s are used to promote students’ engagement in online PBL. Previous research has argued that PBL employs scaffolding extensively, but seldom categorizes the scaffoldings (Hmelo-Silver et al., 2007).

**Figure 6.**
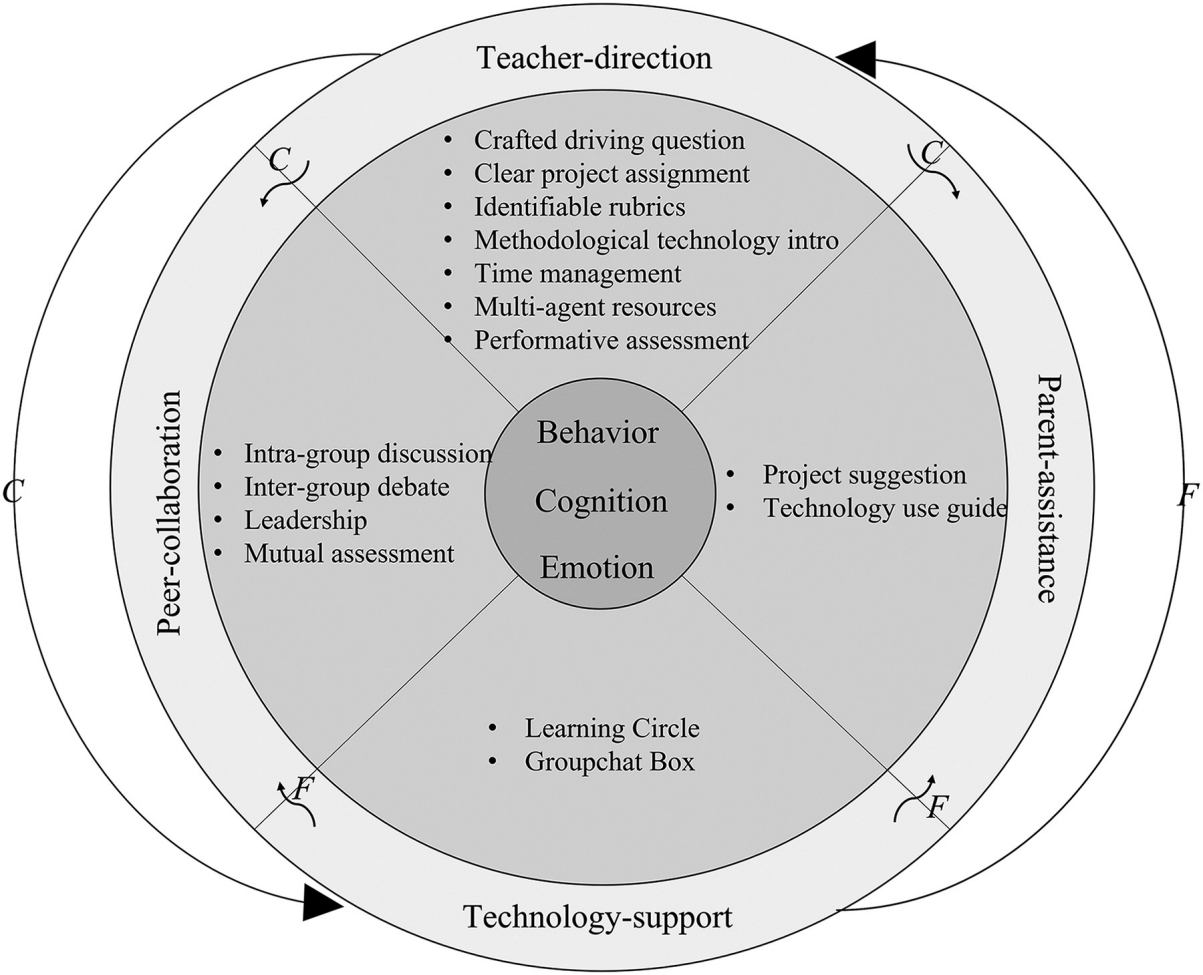
Engaging students in online PBL through appropriate pedagogies. *Note*. C means control. F means facilitate.

Resonating with the previous discussion on the facilitators of students’ online learning (Skinner & Pitzer, 2012) (See Figure 1), the data suggest four fundamental scaffolds: teacher direction, peer collaboration, parent assistance, and technology support. Specific scaffoldings also emerged in the data (Figure 6). Theoretically, this study contributes to the development and discussion of the scaffold(ing)s model for engaging students. Shedding a rare light on scaffold(ing)s that engage students, it goes beyond innovating new pedagogies, but concentrates on applying various scaffold(ing)s pedagogically.
